# The Impact of Math Attitudes and Gender in Future School Choice: A Longitudinal Study Among Italian Students

**DOI:** 10.3390/jintelligence14030038

**Published:** 2026-03-02

**Authors:** Lorenzo Esposito, Irene Tonizzi, Maria Carmen Usai, David Giofrè

**Affiliations:** Department of Education (Disfor), University of Genoa, Corso Andrea Podestà, 2, 16128 Genoa, Italy; irene.tonizzi@edu.unige.it (I.T.); maria.carmen.usai@unige.it (M.C.U.)

**Keywords:** school track choice, gender differences, math self-concept, math anxiety

## Abstract

Previous research indicates that cognitive and affective-motivational factors, along with gender, influence students’ educational choices, especially regarding STEM tracks. However, few longitudinal studies have examined these factors during middle school, a critical stage in shaping future academic trajectories. This study investigated the longitudinal contribution of gender, cognitive abilities, and affective-motivational factors, such as self-concept, math interest, and math anxiety, in predicting students’ school choice between STEM and non-STEM tracks at the end of middle school. Data were collected from 159 Italian students, followed from seventh to eighth grade. Findings indicated that gender and positive attitudes toward math were strongly associated with STEM school choice. Boys were more likely than girls to choose STEM tracks (*b* = 5.048). Higher levels of math self-concept (*b* = 4.848) and interest (*b* = 0.887) significantly predicted the likelihood of choosing a STEM school. These results highlight how gender and affective-motivational factors shape educational pathways during adolescence.

## 1. Introduction

Choosing a school track is an important decision in students’ educational paths. This decision frequently affects the direction of future academic and career choices and can be influenced by a wide range of cognitive, emotional, and contextual variables. Cognitive abilities (Guo et al., 2015; Wang & Degol, 2013) and motivational beliefs about school subjects (Chan, 2022; Eccles & Wang, 2016), such as math, are considered key factors influencing students’ choices. Gender^1^ differences in attitudes and beliefs were addressed as potential factors influencing students’ vocational preferences, such as the choice of STEM paths (Ceci et al., 2009; Fisher et al., 2020; Wang & Degol, 2013).

Only a few studies longitudinally examined the specific contribution of multiple factors, such as math attitudes, cognitive performances, and gender differences, to STEM-related school choices during middle school. This period represents a pivotal stage for shaping students’ academic trajectories and professional aspirations, as attitudes toward mathematics and science developed at this age strongly influence future educational and career decisions (Eccles & Wigfield, 2002; Wang & Degol, 2017). By simultaneously considering these diverse aspects, the present study aims to provide a comprehensive understanding of how early cognitive and emotional factors interplay in shaping STEM educational pathways.

### 1.1. Gender Differences in STEM Paths

Despite the progressive increase in women’s participation in higher education, women continue to be underrepresented in STEM (Science, Technology, Engineering, Mathematics) pathways (Breda et al., 2023; Chan, 2022), such as in math-centered academic tracks (i.e., computer science, engineering). The situation has not changed markedly over the last two decades (Breda et al., 2023). Gender differences in STEM career choices are thought to be the result of the complex interaction of motivational, psychological, and socio-cultural factors that influence individuals’ educational and career choice. When discussing cognitive and academic factors, students’ engagement in STEM pathways was found to heavily depend on their cognitive performance and academic achievements. Evidence from longitudinal large-scale studies shows that adolescents with higher spatial skills are more likely to pursue advanced degrees and careers in STEM fields (Lubinski et al., 2006; Wai et al., 2009). Mathematical ability is also important, since it is highly related to course enrollment and pursuit of higher studies in quantitative areas (Guo et al., 2015; Muenks et al., 2018). Also, general intelligence further contributes to overall academic success and provides a robust predictor of educational outcomes across domains (Esposito et al., 2025; Giofrè et al., 2017). It is worth mentioning that intelligence is not a monolithic construct, rather, it is a multidimensional one. In fact, beyond the general cognitive factor, specific components may have distinct roles in predicting enrollment in curricular tracks. Notably, while verbal ability promotes overall academic performance, children with higher verbal than mathematical or spatial skills might favor the enrollment in humanities or social sciences pathways (Lubinski et al., 2006; Wai et al., 2009). Similarly, earlier achievement in math and science might favor both competence and access to more advanced curricular tracks, thereby shaping the range of future academic opportunities (Guo et al., 2015). 

Among affective factors, math anxiety, which can be defined as the fear of learning and being tested in math, was negatively associated with math performance and with STEM school tracks (Cuder et al., 2024; Daker et al., 2021) and career choice (Ahmed, 2018; Cribbs et al., 2021). Notably, math anxiety seems to contribute to a decline in interest in math subjects, probably leading to different career choices (Živković et al., 2023). Supporting this, it was found that students who pursue STEM pathways generally report lower levels of math anxiety (Daker et al., 2021), and girls tend to experience higher levels than boys (Cuder et al., 2024).

Given the influence of affective factors such as math anxiety on students’ engagement and choices (Donolato et al., 2020), it is also important to consider self-efficacy, defined as students’ belief in their own ability to succeed in a task (Putwain et al., 2013). This belief is thought to be a critical component of educational and career choices, influencing decisions starting in middle school and helping students to pursue STEM fields further (Chan, 2022). Past literature consistently showed that girls tend to report lower levels of self-efficacy than boys (Chan, 2022; Fisher et al., 2020). Self-efficacy is a powerful predictor of educational and career choices, influencing decisions as early as middle school and playing an essential part in STEM route persistence (Cuder et al., 2024; Wang & Degol, 2013). Indeed, a lack of confidence in their abilities can deter girls from pursuing further study in these subjects, regardless of their actual skills or performance (Fisher et al., 2020).

In addition to cognitive and affective factors, it is worth noting that educational and career choices could be strongly influenced by personal and cultural interests and values (Ceci et al., 2009; Eccles & Wang, 2016). In this regard, it was suggested that gender differences arise because women could prefer occupations centered on social connections and altruistic reasons (Ceci et al., 2009; Hill et al., 2010; Lubinski et al., 2001; Wang & Degol, 2013), and they place a high value on work–life balance (Eccles & Wang, 2016; Hill et al., 2010; Wang & Degol, 2013). These cultural factors are especially important considering the so-called “gender-equality paradox”, according to which gender differences in academic performance and STEM aspirations are steady or even widening in increasingly gender-equal and wealthy nations (Balducci et al., 2024; Herlitz et al., 2025). For example, intraindividual strengths, such as boys’ relative advantage in science versus reading, appear to be more prominent in situations with more gender equality (Balducci et al., 2025). This paradox implies that when economic and societal limitations are reduced, gender-specific preferences and cognitive talents may play a more important role in influencing educational trajectories.

### 1.2. Math Attitudes and STEM Choice

Self-concept represents a central dimension of attitudes toward math, and it is crucial for understanding how students develop motivations and make decisions regarding educational and professional pathways related to math and STEM disciplines in general (Aquilina et al., 2024; Wang & Degol, 2013). Math self-concept refers to a broad and stable evaluation of one’s overall math abilities and competence, and it is thought to be influenced by school experiences, social comparisons, and feedback (Arens et al., 2021; Ganley & Lubienski, 2016).

Several studies found a strong association between a positive perception of one’s own abilities in math and a higher tendency to choose tracks and careers in math disciplines (Chan, 2022; Cuder et al., 2024). This association is observed even in adolescence and seems consistent even after controlling for math performance (Eccles & Wang, 2016; Fisher et al., 2020). In other words, math self-concept could play an even stronger role than math performance, highlighting how students’ perceptions of their abilities are more important than their actual performance in influencing STEM choice. Supporting this, several studies found evidence that individuals with a high math self-concept also tend to show lower levels of anxiety, and more positive emotional engagement in math activities (Živković et al., 2023). It was suggested that these factors could facilitate long-term persistence and motivation in learning math and pursuing STEM paths (Ahmed, 2018; Cribbs et al., 2021).

Gender differences in math self-concept are likely to contribute to the observed gap in STEM choices between boys and girls (Fisher et al., 2020; Wang & Degol, 2013). Although girls often show similar or even higher math achievement compared to their male peers, they generally report lower levels of math self-concept (Eccles & Wang, 2016; Fisher et al., 2020). This gap likely emerges early in the school path and tends to increase over time, thus reducing girls’ interest and aspiration toward math and STEM careers (Breda et al., 2023; Charlesworth & Banaji, 2019; Legewie & DiPrete, 2014). These findings could also be interpreted in relation to the internalization of gender stereotypes that often associate math field with boys (Ceci et al., 2009; Charlesworth & Banaji, 2019), leading girls to underestimate their own abilities despite their actual performance. In this regard, recent studies found that math self-concept might play a mediating role between math performance and the decision to pursue STEM pathways (Chan, 2022; Lent et al., 2018).

Math self-concept is also strictly related to other motivational and attitudinal factors (Chan, 2022; Wang & Degol, 2013). According to EVT (Eccles & Wang, 2016), in addition to the self-concept, the choice of an educational path is also influenced by the subjective value attributed to the subject and the enjoyment felt in that subject. A higher math self-concept can enhance the effect of an interest or perceived utility, whereas a lower self-concept can decrease it (Chan, 2022; Lent et al., 2018). This relationship highlights the importance of an integrated framework of affective-motivational processes involved in educational and career choices (Cuder et al., 2024; Wang & Degol, 2013).

### 1.3. Aim and Hypotheses

Despite the extensive literature on gender differences in mathematics, research examining cognitive and affective predictors simultaneously within a longitudinal design remains rare. This integrative approach is essential to understand whether STEM trajectories are mainly driven by abilities or self-beliefs. In addition, recent studies have highlighted a “gender-equality paradox”, where gender differences in academic strengths and STEM aspirations appear stable or even more pronounced in more gender-equal and wealthy countries (Balducci et al., 2024; Herlitz et al., 2025). Exploring these dynamics in the Italian context is particularly meaningful since Italy presents a distinct cultural context compared to the highly gender-equal countries typically associated with this paradox, allowing for a valuable contribution on how cognitive and affective factors shape educational choices (Balducci et al., 2025). Therefore, this study aims to assess the longitudinal impact of cognitive factors (i.e., intelligence, working memory, inhibitory control), affective-motivational variables (self-concept, interest in math, and math anxiety), and gender on students’ school track choices at the end of middle school. Based on past research, we investigated whether and to what extent these factors, assessed in both 7th and 8th grade, are associated with students’ attitudes and choices between STEM and non-STEM school tracks. Specifically, we investigated if and to which extent cognitive and affective factors predict STEM school choice, and if gender remains a key predictor after controlling for these variables. Since previous studies showed that gender and affective-motivational factors strongly influence students’ school and career choices, we expect that gender and positive attitudes toward math, such as self-concept and interest, will be associated with students’ choices. Additionally, we expect that higher cognitive performance and prior achievement in math will increase the likelihood of choosing a STEM track. Specifically, we expect boys to be more likely to choose a STEM track compared to girls, and the probability of choosing a STEM track to increase with more positive attitudes.

## 2. Materials and Methods

### 2.1. Sample

The sample included 197 middle school students recruited from three schools in Genoa (Italy), and were longitudinally assessed in 7th and 8th grade (T1 and T2), approximately one year apart. Participants with genetic syndromes, intellectual disabilities, or developmental and neurological disorders were excluded from the study. The final sample consisted of 159 students at T1 (74 males, 85 females), aged 12.94 years (*SD* = 0.45) or 155.32 months (*SD* = 5.37), and 154 students at T2 (74 males, 85 females), aged 13.94 years (*SD* = 0.45) or 167.28 months (*SD* = 5.38). The study received approval from the Ethics Committee of the University of Genoa (protocol code 2024.13, 20 February 2024). Written informed consent was obtained from all participants or their legal guardians prior to participation, in accordance with the applicable ethical guidelines.

### 2.2. Procedure

The study was conducted over two longitudinal time periods (T1 and T2), between February and May of the 7th- and 8th-grade school years. In each time period, participants were assessed in distinct sessions through collective classroom and individual assessments. In the first session, participants were assessed through paper-and-pencil tests on general intelligence and math anxiety. Participants were also asked to complete a brief questionnaire about vocational aspects, such as positive attitudes toward math and future school choice. Working memory and inhibitory control were assessed through computerized tasks in the second session. Math achievement was assessed through a paper-and-pencil test administered in a third session. Each session lasted approximately one hour.

### 2.3. Measures

#### 2.3.1. Math Achievement

A standardized math test (Amoretti et al., 1997) was used to assess math achievement. The test is based on the objectives of the Italian national curriculum and provides a year-specific measure of math competence. According to curricular guidelines, students are expected to demonstrate understanding of arithmetic, geometry, and basic statistics. The total score was calculated as the sum of correct responses (Cronbach’s alpha, *α* = .892).

#### 2.3.2. Intelligence

The Primary Mental Abilities (PMA) tests evaluate different cognitive domains (Thurstone, 1937), and scores are computed as the total number of correct responses in each subtest. Three main cognitive abilities were assessed: spatial, verbal, and reasoning. In the spatial subtest, participants had to identify identical figures among six rotated and mirrored alternatives. This subtest consisted of 20 items to be completed in 5 min (*α* = 0.919). In the verbal subtest, participants had to select synonyms for given words among four options, such as choosing “to look” as a synonym for “to watch”. The subtest consisted of 50 items to be completed in 4 min (*α* = 0.937). In the reasoning subtest, participants were required to complete letter sequences by selecting the logically correct letter, for instance, identifying “h” as the next letter in sequences like “aab”, “ccd”, “eef”, and “gg”. This test included 30 items to be completed within 6 min (*α* = 0.919).

#### 2.3.3. Working Memory

Four measures of WM were collected: two for verbal WM and two for visuo-spatial WM. These tasks were adapted from the previous literature (Esposito et al., 2025; Giofrè et al., 2017), and WM capacity was calculated as the total number of correct responses. Verbal WM was assessed using a backward word span task and a verbal dual task. In the backward span task, participants listened to lists of words which gradually increased in length from 2 to 8 items, with two trials per span (70 words in total) and were asked to repeat the words in reverse order (*α* = 0.882). In the verbal dual task, participants listened to lists of four words, with the number of lists increasing from 2 to 6 across trials. Each span included two trials, with 160 words presented in total and with 40 words to recall. While listening, participants were instructed to press the spacebar when they heard an animal name and to remember the last word of each list. At the end of each trial, they had to recall the final words in the correct order. The lists did not include math or geometric words (*α* = 0.922). Both verbal tasks presented one word every two seconds, and participants practiced with a two-word example before starting. A score of 1 was given for each correct response and 0 for incorrect responses. Visuo-spatial WM was assessed with a backward matrices task and a visuo-spatial dual task, both using a 4 × 4 grid. In the backward matrices task (*α* = 0.942), participants viewed sequences of black squares that increased in length from 2 to 8, with two trials per span (70 squares total), and were asked to reproduce the sequence in reverse order. In the dual task, each grid included gray squares, followed by the sequential presentation of three black dots. Participants had to press the spacebar when a dot appeared in a gray square and remember the position of the last dot in each grid. After viewing all grids for that trial, they had to recall the positions of the final dots in the correct order (*α* = 0.940). The number of grids increased from 2 to 6 across trials, with two trials per span, with 120 dots presented in total and 40 dots to recall. Stimuli in both visuo-spatial tasks were presented at a rate of one every two seconds, and participants practiced with a two-item example before starting. Correct responses were scored as 1, and incorrect ones as 0.

#### 2.3.4. Inhibitory Control

Three computerized tasks were used to measure IC: Stroop, Flanker, and Simon tasks. These tasks were customized for children based on prior versions used with adults (Burgoyne et al., 2023). To ensure that participants understood the instructions, they were asked to correctly respond at least two times in a training block before moving on to the testing phase. Feedback was only supplied at the end of the training block. Each task was designed to increase interference at the response level, and performance was measured as the sum of correct responses minus the sum of incorrect ones. In the Stroop task (*α* = 0.959), children were shown a target word (e.g., “RED” or “BLUE”) in red or blue display color, with two alternatives below it. The participant was required to respond based solely on the display color of the target word, disregarding its semantic content and the display color of the response options. For example, if the word “RED” appeared in blue, the right choice was “BLUE” regardless of the color used. In the Flanker task (*α* = 0.941), children were shown five-arrow sequences (e.g., > > < > >) and had to identify the direction of the flanking arrows while ignoring the central arrow. They also had to focus on the central arrow in the response alternatives while disregarding the flanking arrows. For example, the target < < > < < required the participant to select the choice with a central arrow pointing left. In the Simon task (*α* = 0.955), children were shown an arrow pointing left or right and had to select the response choice labeled “LEFT” or “RIGHT” based on the arrow’s direction. They ignored the spatial location of the arrow and the response alternatives on the screen. For example, if the arrow pointed to the left, the correct response was “LEFT”, regardless of its position on the screen.

#### 2.3.5. Math Anxiety

The Italian standardized version of the Abbreviated Mathematics Anxiety Scale (Caviola et al., 2017) was used to assess MA. This self-reported questionnaire includes 9 items assessing fear of learning math content and being tested in math. Participants rated how anxious they felt in math-related situations on a scale of 1 to 5. The total score is the sum of all items, and a high score indicates a higher level of anxiety toward math (*α* = 0.853).

#### 2.3.6. School Choice and Attitudes Toward Math

A brief self-report questionnaire was administered to participants, to examine their school choices. In Italy, students are free to choose their high school and educational program, and they are typically required to make their decisions before the end of middle school, so participants were asked to provide the name of their high school and the educational program they had chosen. Participants were also asked to rate their perceived competence (e.g., “How well do you think you are doing in math?”) and their interest (e.g., “How much do you like math?”) in math on a scale of 1 to 5. This information was collected at both T1 and T2, to track possible changes in students’ attitudes over time. It is worth mentioning that self-concept is considered as the broad evaluation of one’s own ability, whereas self-efficacy is defined as the confidence in successfully accomplishing specific tasks. We acknowledge their conceptual overlap, and although they are strongly related, we treated them as distinct constructs, aiming to capture distinct nuances of students’ attitudes.

### 2.4. Data Analysis

Analyses were performed in R (R Core Team, 2024, version 4.4), using RStudio (version 2024.12) as the IDE (RStudio Team, 2024). Before running the analyses, school choices were clustered a priori into two main categories, non-STEM and STEM, following criteria from past studies (Cuder et al., 2024). The total score for each measure was calculated (see Measures), then a composite score for each variable was calculated. Math achievement was measured as the number of correct responses on the math test, while math anxiety score was calculated as the sum of responses. Composite scores for intelligence, WM, and IC were calculated from subtest totals. All scores were converted into z-scores before analyses. First, a series of correlation analyses was conducted to examine the relationships among variables. Subsequently, to investigate the contribution of affective and cognitive factors, and gender on school choice, we conducted a series of logistic regressions following a hierarchical approach with the school choice as the response variable. Logistic regression was used because it is specifically designed to model binary outcome variables, allowing estimation of the probability of an event occurring. The hierarchical method was used to examine the incremental contribution of each set of predictors (e.g., cognitive, affective, and gender) to the model. School choice was coded as 0 = non-STEM and 1 = STEM, while gender was coded with girls as the first group and boys as the second group. Odds ratios were reported to facilitate interpretation of the results. Cook’s distance, with a threshold of 3/*n*, was used to identify potential influential cases. Cases exceeding the critical Cook’s distance threshold were removed to ensure the robustness of the models. To assess the goodness-of-fit of the logistic regression models, the Nagelkerke pseudo-*R*^2^ was considered, which indicates the proportion of variance explained by the model (Nagelkerke, 1991). Relative model fit was evaluated using AIC (Akaike Information Criterion) and BIC (Bayesian Information Criterion), with lower values indicating better model fit (Burnham & Anderson, 2002).

## 3. Results

### 3.1. Preliminary Analysis

Descriptive statistics are shown in Table 1. The results showed a positive and moderate-to-strong correlation between the measures collected at T1 and T2, suggesting the stability of our variables across grades (see Table 1; a complete list of correlations can be found in Table S1 of the Supplementary Materials). School choice was positively correlated with all other variables, except MA, suggesting that the probability of choosing a STEM school track depends on both cognitive and affective factors. On the one hand, the probability of choosing a STEM school track increases with math achievement and other cognitive abilities such as intelligence, WM, and IC, although effect sizes show weak correlations; on the other hand, the probability of choosing a STEM school track decreases as MA increases, suggesting that the fear of learning and being tested in math could discourage enrollment in a STEM-track school. Notably, school choice was strongly and positively associated with the self-concept and interest in math at both T1 and T2, suggesting that these positive attitudes toward math could play an important role in future school choice (see Table 1).

### 3.2. Hierarchical Logistic Regressions

To examine the influence of affective and cognitive factors, as well as gender, on school choice, we performed a series of hierarchical logistic regression analyses (see Table 2). In Model 1, we included only gender to assess the direct effect of gender on school track choice. This model suggested that gender has a strong influence on the probability of choosing a STEM track school, *b* = 1.660, *OR* = 5.259, *p* < .001. This model explained about 18% of the variance. Subsequently, in Model 2, we added the cognitive factors at T1 thought to affect the school choice: math achievement, intelligence, WM, and IC. Gender was the only significant predictor, even after controlling for these variables, *b* = 1.292, *OR* = 3.639, *p* < .01. In Model 3, we added the affective factors at T1 thought to affect school choice, along with gender: MA, self-concept, and interest in math. Results showed that gender, *b* = 1.601, *OR* = 4.956, *p* < .01, together with self-concept, *b* = 1.507, *OR* = 4.513, *p* < .01, and interest, *b* = 0.977, *OR* = 2.657, *p* < .01, were strong predictors of school choice. In Model 4, we added all variables at T1, including gender to test the joint contribution of cognitive and affective factors. Again, gender, *b* = 1.267, *OR* = 3.550, *p* < .05, self-concept, *b* = 1.708, *OR* = 5.518, *p* < .01, and interest, *b* = 0.887, *OR* = 2.429, *p* < .05, were strong predictors of school choice. In Model 5, we tested the cognitive predictors at T2 together with gender, the latter was again the only significant predictor, *b* = 1.733, *OR* = 5.657, *p* < .01. In Model 6, we added to gender the affective factors at T2, and we found again that gender, *b* = 1.907, *OR* = 6.731, *p* < .01, self-concept, *b* = 1.17, *OR* = 3.223, *p* < .01, and interest, *b* = 1.002, *OR* = 2.725, *p* < .01, were significant predictors of school choice. Subsequently, we tested the joint contribution of cognitive and affective factors at T2 in Model 7, and we found that only gender, *b* = 1.944, *OR* = 6.987, *p* < .01, significantly predicted school choice. In Model 8, we tested the contribution of all factors measured at both T1 and T2. Results showed that gender, *b* = 2.488, *OR* = 12.031, *p* < .05, and self-concept at T1, *b* = 3.173, *OR* = 23.870, *p* < .05, were significant predictors of school choice. In Model 9, we controlled also for the school choice made at T1 to test possible autoregressive effects. Again, we found that gender, *b* = 5.048, *OR* = 155.678, *p* < .05, and self-concept at T1, *b* = 4.848, *OR* = 127.433, *p* < .05, were strong predictors of school choice, even after controlling for school choice at T1. This model showed good fit indices, Nagelkerke *R*^2^ = .926, *AIC* = 59.473, *BIC* = 95.906.

In summary, our findings show that gender is among the most important factor impacting school choice, with males being more likely to choose a STEM program. Along with gender, students’ self-concept and interest in math were significant predictors. In contrast, cognitive performances had minimal influence on school decisions. Overall, these findings indicate that what students perceive and feel about math is more important than their actual performance when it comes to determining their educational pathway.

## 4. Discussion

### 4.1. Findings on Gender

This study explored how gender and cognitive and affective-motivational factors influence students’ future educational choices as they move from middle to high school. The integration of cognitive and non-cognitive elements, representing a gap in intelligence research, is a major contribution of this work. This study looks at what intelligence predicts when combined with other predictors, like gender and math self-concept. Our results show that once affective-motivational factors are included, intelligence is not enough to explain school track choices. This is a significant finding as it offers a clearer picture of intelligence as a predictor. The study’s longitudinal design and analysis of affective-motivational factors (such as math self-concept, interest, and MA) as well as cognitive skills (such as math achievement, intelligence, WM, and IC) demonstrate the role that gender and math self-concept play in predicting the likelihood of choosing a STEM track over a non-STEM track a year later. Although cognitive performances such as mathematics achievement, intelligence, WM, and IC were considered, their influence on school track choice was minimal compared to gender and affective-motivational factors, suggesting that students’ perceptions and attitudes toward math are more crucial than their real skills. These cognitive factors showed moderate stability over time, implying that children’s underlying skills remained quite steady throughout middle school. This suggests that, while cognitive abilities might be necessary for STEM accomplishment, they are insufficient to account for school track choices. Therefore, this study extends prior research by focusing on middle school and how gender differences and math attitudes influence students’ choices.

Gender consistently emerged as a strong predictor of school track choice, with boys being significantly more likely to pursue STEM pathways compared to girls. This finding suggests that, even when cognitive skills are accounted for, socio-cultural expectations may remain the dominant influence on STEM choice. Furthermore, the gender gap was consistent across all models, even when controlling for cognitive and affective-motivational factors. These findings suggest that differences in students’ perceptions of their competence and interest in math likely cannot fully account for the impact of gender on educational decisions. According to this finding, the gender gap in STEM is not just due to differences in skills or interests. It likely reflects cultural norms and potentially institutional factors that influence students’ beliefs and decisions. Such findings are in line with previous literature showing that gender disparities in STEM engagement often persist beyond explainable variance in motivation or ability (Nollenberger et al., 2016; Wang & Degol, 2016), and align with research indicating that unmeasured sociocultural or identity-related factors continue to influence educational aspirations (Brand & Xie, 2010).

### 4.2. Findings on Self-Concept

Math self-concept, defined as a broad and consistent belief about one’s math competence (Arens et al., 2021), predicted STEM track choice even after controlling for prior choices and other factors. This suggests that early self-beliefs in math could have a direct effect on future educational decisions, increasing the gender gap in STEM fields. These findings are in line with the Social Cognitive Career Theory (SCCT), according to which self-efficacy is important for educational and career decisions (Lent et al., 2018). Similarly, according to the Expectancy-Value Theory (EVT; Eccles & Wang, 2016), educational decisions are influenced by both expectations of success and the subjective value assigned to a certain domain.

A higher level of math self-concept may increase students’ confidence in their abilities as well as the importance they attribute to mathematics, which in turn increases the likelihood of choosing a STEM track. Conversely, a lower level of math self-concept could lead students to disengage from it and prefer non-STEM fields. Importantly, prior research showed that math self-concept is not only affected by negative or positive experiences, but also by teacher feedback and gender stereotypes (Ganley & Lubienski, 2016; Charlesworth & Banaji, 2019). Girls may internalize stereotypes that gradually weaken their confidence and dissuade them from pursuing math-related studies and careers, even when they are fully capable (Ceci et al., 2009; Fisher et al., 2020). Importantly, our study’s longitudinal design demonstrates that math self-concept measured in 7th grade remains a strong predictor of STEM track choice one year later, even after controlling for earlier choices. This demonstrates that self-beliefs formed in early adolescence can have a long-term impact on educational and career pathways. Supporting this, we found moderate-to-strong correlations between variables assessed at T1 and T2, demonstrating the strong stability of both cognitive and affective characteristics over two school years, underscoring their crucial role in shaping students’ academic paths. Interestingly, while cognitive abilities showed moderate stability over time, their direct effect on school track selection was limited, highlighting the pivotal importance of motivational factors and gender. Moreover, math self-concept measured in 7th grade significantly predicted STEM track choice one year later, even after controlling for prior choices, suggesting that early self-perceptions and beliefs can have a lasting impact on educational choices.

### 4.3. Limits and Future Research

While this study offers valuable insights, certain limitations should be acknowledged. First, math self-concept and interest were assessed using single-item measures. Although this approach is frequently utilized in longitudinal research, in this study, it might limit sensitivity and reliability compared to scales with multiple items. Consequently, these results should be interpreted with caution, and future studies should aim to validate more robust measures. Importantly, self-report instruments for affective-motivational variables (e.g., self-efficacy, interest) could be prone to social desirability bias or to subjective interpretations of one’s own experiences. To increase validity, future research could use implicit measurement techniques. Second, the sample size was relatively modest, and this may have limited statistical power, particularly for detecting small effect sizes, and restricts the generalizability of the findings. In this regard, it is worth mentioning that in Model 7, interest in mathematics measured at T2 approached the statistical significance threshold, *b* = 0.818, 95% CI [0.01; 1.72], *p* = .057. It is plausible that this variable has a meaningful impact on the outcome, which was difficult to detect reliably with a limited sample size. Third, this study did not measure environmental factors such as parental context, teacher-related factors (e.g., instructional clarity, achievement expectations), or broader school characteristics. Our findings are limited by the lack of these variables, which could partially explain the gender predictive effect that persists even after adjusting for cognitive and affective factors. Future research should examine this interaction using longitudinal designs, especially evaluating children at younger ages, since these contextual factors could already be strongly related to the investigated variables. Additionally, future research could explore the effectiveness of clinical, or school interventions aimed at fostering self-concept and interest in math to promote more equitable access to STEM pathways. Fourth, as for the role of MA, it was included as a direct predictor and we did not test more complex models. Therefore, future research could extend this work by testing more complex structural models to explore potential mediating or moderating roles of anxiety in the relationship between cognitive factors and school choice. Finally, the final models (e.g., Models 8 and 9) included a large number of predictors compared to the sample size, which could have led to a possible overfitting risk, suggested by extremely high odds ratios and wide confidence intervals. Therefore, results should be interpreted with caution, and future research could aim to replicate these findings with a larger sample, tested several times in a longitudinal design.

### 4.4. Practical Implications

These findings have significant consequences for educational practices. While cognitive abilities such as mathematical achievement, intelligence, working memory, and inhibitory control are not direct predictors of STEM track choice, they do provide a fundamental and necessary skill required for success and accomplishment in these domains. This suggests that interventions focused exclusively on enhancing cognitive performance may be insufficient to improve interest in STEM. Instead, integrating cognitive development support with measures to improve math self-concept and enthusiasm may be more beneficial in encouraging children’s persistence in STEM fields. Recognizing the minimal direct influence of cognitive capabilities highlights the importance of addressing socio-cultural and motivational elements that have a greater impact on students’ educational decisions.

## 5. Conclusions

This study examines how gender and affective-motivational factors impact school track choices in middle school students. We speculate that, despite being important for success in both STEM and non-STEM fields, cognitive abilities are less directly involved in choosing future educational and career paths. Conversely, gender and early measures of self-concept and math interest strongly predicted the choice of STEM tracks. These findings highlight the importance of early motivational factors on students’ future educational decisions.

## Figures and Tables

**Table 1:** Correlations.

	Gender	Math	Intelligence	WM	IC	MA	Self-Concept	Interest	Choice
Gender	-	.149	.049	.122	.16	-.154	.319 ***	.168 *	.337 ***
Math	.168 *	.663 ***	.531 ***	.336 ***	.358 ***	-.332 ***	.352 ***	.305 ***	.215 *
Intelligence	.011	.572 ***	.787 ***	.259 **	.433 ***	-.239 **	.247 **	.165 *	.166 *
WM	.205 *	.473 ***	.462 ***	.385 ***	.291 ***	-.056	.004	.066	.178 *
IC	.191 *	.437 ***	.505 ***	.486 ***	.778 ***	-.21 *	.268 **	.108	.174 *
MA	−.281 ***	−.247 **	−.173 *	−.331 ***	−.34 ***	.651 ***	−.409 ***	−.423 ***	−.136
Self-concept	.174 *	.32 ***	.277 ***	.285 **	.385 ***	−.433 ***	.736 ***	.604 ***	.284 ***
Interest	.166 *	.244 **	.081	.179 *	.118	−.293 ***	.619 ***	.714 ***	.325 ***
Choice	.369 ***	.221 *	.166	.267 **	.186 *	−.294 ***	.487 ***	.505 ***	.425 ***

Note. Gender is coded as: F = 0, M = 1; Choice is coded as: non-STEM = 0, STEM = 1; WM = Working memory; IC = Inhibitory control; MA = Math anxiety; Values above the diagonal indicate T1 correlations; values below the diagonal indicate T2. Autoregressive correlations are reported on the diagonal; * *p* < .05, ** *p* < .01, *** *p* < .001.

**Table 2:** Unstandardized solutions of logistic regressions.

Model	*R* ^2^	*AIC*	*BIC*	Predictor(s)	*b*	*SE*	95% CI	*OR*	*p*
1	.184	140.127	145.634						
				Gender	1.66	0.426	[0.85; 2.53]	5.259	<.001
2	.367	126.033	141.543						
				Gender	1.292	0.459	[0.41; 2.22]	3.639	<.01
				Math (T1)	0.533	0.281	[0; 1.11]	1.704	.058
				Intelligence (T1)	−0.008	0.299	[−0.6; 0.58]	0.992	.979
				WM (T1)	−0.111	0.261	[−0.64; 0.4]	0.895	.671
				IC (T1)	0.075	0.271	[−0.46; 0.61]	1.078	.783
3	.620	97.982	111.707						
				Gender	1.601	0.543	[0.56; 2.71]	4.956	<.01
				MA (T1)	0.547	0.332	[−0.08; 1.23]	1.729	.099
				Self-concept (T1)	1.507	0.448	[0.7; 2.48]	4.513	<.01
				Interest (T1)	0.977	0.352	[0.31; 1.71]	2.657	<.01
4	.663	93.495	116.288						
				Gender	1.267	0.616	[0.09; 2.54]	3.55	<.05
				Math (T1)	−0.27	0.433	[−1.14; 0.58]	0.763	.533
				Intelligence (T1)	−0.03	0.352	[−0.73; 0.67]	0.971	.933
				WM (T1)	0.303	0.326	[−0.34; 0.97]	1.354	.354
				IC (T1)	−0.142	0.343	[−0.84; 0.53]	0.867	.678
				MA (T1)	0.449	0.39	[−0.3; 1.26]	1.567	.250
				Self-concept (T1)	1.708	0.53	[0.77; 2.87]	5.518	<.01
				Interest (T1)	0.887	0.407	[0.12; 1.74]	2.429	<.05
5	.506	111.737	127.06						
				Gender	1.733	0.514	[0.76; 2.79]	5.657	<.01
				Math (T2)	0.386	0.322	[−0.24; 1.04]	1.471	.231
				Intelligence (T2)	−0.023	0.342	[−0.71; 0.65]	0.977	.947
				WM (T2)	0.792	0.403	[0.06; 1.65]	2.207	.050
				IC (T2)	−0.347	0.322	[−1; 0.28]	0.707	.281
6	.610	99.922	113.603						
				Gender	1.907	0.559	[0.86; 3.07]	6.731	<.01
				MA (T2)	0.149	0.345	[−0.53; 0.84]	1.161	.665
				Self-concept (T2)	1.17	0.433	[0.37; 2.08]	3.223	<.01
				Interest (T2)	1.002	0.373	[0.31; 1.79]	2.725	<.01
7	.728	86.376	108.569						
				Gender	1.944	0.691	[0.65; 3.4]	6.987	<.01
				Math (T2)	−0.24	0.421	[−1.1; 0.58]	0.786	.568
				Intelligence (T2)	0.509	0.454	[−0.37; 1.44]	1.664	.263
				WM (T2)	0.123	0.486	[−0.84; 1.11]	1.131	.799
				IC (T2)	−0.269	0.431	[−1.13; 0.59]	0.764	.532
				MA (T2)	−0.587	0.454	[−1.52; 0.29]	0.556	.196
				Self-concept (T2)	0.806	0.53	[−0.18; 1.94]	2.238	.129
				Interest (T2)	0.818	0.429	[0.01; 1.72]	2.266	.057
8	.846	73.245	108.52						
				Gender	2.488	1.118	[0.51; 5.06]	12.031	<.05
				Math (T1)	−0.568	0.828	[−2.28; 1.04]	0.567	.492
				Math (T2)	−0.418	0.73	[−1.96; 1.01]	0.659	.568
				Intelligence (T1)	−0.201	0.882	[−2.03; 1.58]	0.818	.820
				Intelligence (T2)	0.74	1.029	[−1.25; 2.92]	2.097	.472
				WM (T1)	−0.231	0.539	[−1.38; 0.82]	0.794	.669
				WM (T2)	−0.607	0.918	[−2.58; 1.08]	0.545	.508
				IC (T1)	−0.293	0.778	[−1.87; 1.28]	0.746	.707
				IC (T2)	0.31	0.786	[−1.31; 1.89]	1.363	.693
				MA (T1)	0.175	0.788	[−1.36; 1.81]	1.192	.824
				MA (T2)	0.423	0.711	[−0.94; 1.94]	1.526	.552
				Self-concept (T1)	3.173	1.385	[0.87; 6.54]	23.87	<.05
				Self-concept (T2)	1.015	0.873	[−0.6; 2.95]	2.761	.245
				Interest (T1)	0.123	0.878	[−1.73; 1.84]	1.131	.888
				Interest (T2)	0.389	0.62	[−0.82; 1.69]	1.475	.531
9	.926	59.473	95.906						
				Gender	5.048	2.285	[1.41; 11.03]	155.678	<.05
				Math (T1)	−0.669	1.144	[−3.09; 1.58]	0.512	.559
				Math (T2)	2.107	1.413	[−0.19; 5.67]	8.222	.136
				Intelligence (T1)	0.358	1.567	[−2.79; 3.98]	1.43	.819
				Intelligence (T2)	2.248	1.546	[−0.46; 5.92]	9.47	.146
				WM (T1)	−0.254	1.164	[−2.85; 2.11]	0.776	.827
				WM (T2)	−0.302	1.058	[−2.69; 1.76]	0.739	.775
				IC (T1)	−2.401	1.502	[−6.42; 0.02]	0.091	.110
				IC (T2)	−0.559	1.195	[−3.39; 1.62]	0.572	.640
				MA (T1)	−0.184	1.312	[−3.12; 2.76]	0.832	.888
				MA (T2)	−0.23	1.351	[−3.35; 2.58]	0.795	.865
				Self-concept (T1)	4.848	2.009	[1.69; 10.19]	127.433	<.05
				Self-concept (T2)	−1.012	1.36	[−4.17; 1.6]	0.363	.457
				Interest (T1)	−1.364	1.722	[−5.6; 1.61]	0.256	.428
				Interest (T2)	1.144	1.226	[−1.1; 4.36]	3.139	.351
				Choice (T1)	2.989	1.68	[0.13; 7.31]	19.866	.075

Note. Gender is coded as: F = 0, M = 1; Choice is coded as: non-STEM = 0, STEM = 1; WM = Working memory; IC = Inhibitory control; MA = Math anxiety.

## Data Availability

The data presented in this study are available in OSF at https://osf.io/g82bp/overview?view_only=d5a1ce43a24b487db777e9abda61ff1a (accessed on 17 November 2025).

## Note

1 In this paper, we use the term “gender” because this variable was assessed via self-report.

## Supplementary Materials

The following supporting information can be downloaded at https://www.mdpi.com/article/10.3390/jintelligence14030038/s1; Table S1: Correlations between all variables.
