# Supplementary material for: The Impact of Math Attitudes and Gender in Future School Choice: A Longitudinal Study Among Italian Students
Source: J Intell. 2026 Mar 2;14(3):38. doi: 10.3390/jintelligence14030038 (PMC13027840; doi:10.3390/jintelligence14030038)
Supplement: Supplementary file 1 [file jintelligence-14-00038-s001.zip › jintelligence-4027793-supplementary.pdf]

The impact of math attitudes and gender in future school choice: a longitudinal study

Supplementary materials

Table S1. Correlations between all variables.

	Genere_T1	Math_T1	Math_T2	g_T1	g_T2	WM_T1	WM_T2	EF_T1	EF_T2	MA_T1	MA_T2	SC_T1	SC_T2	IN_T1	IN_T2	STEM_T1
Genere_T1	1															
Math_T1	.149	1														
Math_T2	.168	.663	1													
g_T1	.049	.531	.561	1												
g_T2	.011	.45	.572	.787	1											
WM_T1	.122	.336	.33	.259	.259	1										
WM_T2	.205	.419	.473	.398	.462	.385	1									
EF_T1	.16	.358	.461	.433	.33	.291	.441	1								
EF_T2	.191	.313	.437	.506	.505	.234	.486	.778	1							
MA_T1	-.154	-.332	-.306	-.239	-.229	-.056	-.365	-.21	-.265	1						
MA_T2	-.281	-.24	-.247	-.248	-.173	-.139	-.331	-.247	-.34	.651	1					
SC_T1	.319	.352	.253	.247	.177	.004	.192	.268	.222	-.409	-.48	1				
SC_T2	.174	.325	.32	.339	.277	.097	.285	.351	.385	-.418	-.433	.736	1			
IN_T1	.168	.305	.22	.165	.091	.066	.231	.108	.063	-.423	-.383	.604	.526	1		
IN_T2	.166	.195	.244	.174	.081	.083	.179	.092	.118	-.287	-.293	.497	.619	.714	1	
STEM_T1	.337	.215	.19	.166	.163	.178	.18	.174	.162	-.136	-.169	.284	.183	.325	.25	1
STEM_T2	.369	.213	.221	.167	.166	.058	.267	.159	.186	-.206	-.294	.537	.487	.502	.505	.425

Note. Genere_T1 / Genere_T2 = Gender at Time 1 / Time 2; Math_T1 / Math_T2 = Mathematics performance at Time 1 / Time 2; g_T1 / g_T2 = Intelligence at Time 1 / Time 2; WM_T1 / WM_T2 = Working memory at Time 1 / Time 2; EF_T1 / EF_T2 = Inhibitory control at Time 1 / Time 2; MA_T1 / MA_T2 = Math anxiety at Time 1 / Time 2; SC_T1 / SC_T2 = Mathematics self-concept at Time 1 / Time 2; IN_T1 / IN_T2 = Interest in mathematics at Time 1 / Time 2; STEM_T1 / STEM_T2 = School track choice at Time 1 / Time 2.
